# Approaches to Offering Hepatitis C Treatment at Syringe Services Programs in the United States: A Scoping Review

**DOI:** 10.1093/ofid/ofaf211

**Published:** 2025-04-08

**Authors:** Theodore Yoder, Kyoko Hirose, Judith I Tsui, Judith Feinberg, Holly Hagan, Mai T Pho, Sarah E Rowan

**Affiliations:** University of Colorado School of Medicine, Aurora, Colorado, USA; University of Chicago Medical Center, Chicago, Illinois, USA; University of Washington School of Medicine, Seattle, Washington, USA; West Virginia University School of Medicine, Morgantown, West Virginia, USA; New York University School of Global Public Health, New York, New York, USA; University of Chicago Medical Center, Chicago, Illinois, USA; University of Colorado School of Medicine, Aurora, Colorado, USA; Public Health Institute at Denver Health, Denver, Colorado, USA

**Keywords:** harm reduction, hepatitis C, syringe service programs, telehealth

## Abstract

**Background:**

Innovative strategies are required to treat hepatitis C (HCV) among people who inject drugs (PWID). Integration of HCV treatment in syringe services programs (SSPs) may improve access, although multiple implementation challenges have been described.

**Methods:**

We performed a scoping review of published models of HCV treatment integrated in SSPs in the United States.

**Results:**

We found 13 articles including randomized controlled trials, observational studies, cohort analyses, and qualitative analyses that described a variety of approaches to integration that produced significant improvements in treatment initiation and cure compared with a referral-based standard of care. Variations in delivery models (mobile unit vs brick-and-mortar sites), provider location (on-site vs telehealth), pretreatment evaluation, pharmacy access, supportive services (eg, peer navigation) and funding were described.

**Conclusions:**

Expansion of these models in the United States would not only contribute to HCV elimination but also create opportunities for the provision of other key healthcare services to this important population.

The incidence of hepatitis C (HCV) infection has risen dramatically over the past decade, driven largely by an increase in the number of people who inject drugs (PWID), as a result of the opioid epidemic [1, 2]. As injection drug use is the primary risk factor for HCV transmission, interventions that improve access to screening and treatment for PWID are considered high priority in both national treatment guidelines and the Department of Health and Human Services Viral Hepatitis National Strategic Plan [3, 4]. Despite this recognition and ample evidence of high treatment efficacy in this population [5–9], HCV treatment remains disproportionately low among PWID [10–12].

To address this disparity and crucial public health issue, innovative approaches to improve access and uptake of HCV treatment for PWID are needed. Interventions aimed at serving this population must consider their specific needs, which are often confounded by systemic and socioeconomic factors [13]. Studies have shown that PWID prefer care in locations that are affirming and convenient [14–16]. Many programs outside the United States have demonstrated successful integration of HCV treatment with syringe service programs (SSPs) [17–19]. Examples of integrated models of HCV care with SSPs in the United States are more limited but have also shown benefit and promise [20]. The standard of care for HCV treatment for PWID includes referrals to gastroenterologists, infectious diseases specialists, and HCV-trained primary care providers in traditional clinic settings [21]. This clinic-based approach to HCV treatment has not met the needs of many PWID, with HCV treatment initiation rates of 5.5%–26% among PWID in various reports, and cure rates that are even lower [10–12]. Not only do traditional, typically high-threshold treatment models present scheduling and transportation challenges, they may also be staffed by providers who have limited experience working with PWID or other individuals with substance use disorders [13].

The unique aspects of the US health care system necessitate a better understanding of how SSP-based HCV treatment programs are conducted. We aimed to identify and summarize the published literature describing HCV treatment through partnerships that provided the complete HCV care continuum, including screening, pretreatment evaluation, treatment initiation, treatment completion, and determination of sustained virologic response 12 weeks after the end of treatment (SVR12) at the SSP. We summarize various approaches to SSP-based HCV treatment including program structure, financial coverage, location of the prescribing clinician, pretreatment evaluation, medication delivery and storage, and wrap-around support services.

## METHODS

### Study Design

We conducted a scoping review of published, peer-reviewed literature on approaches to integrating HCV treatment at US SSPs. This review was guided by the Preferred Reporting Items for Systematic Reviews and Meta-Analyses (PRISMA) guidelines.

### Search Strategy

We utilized 3 online databases, PubMed, Embase, and Web of Science, to search for all papers relating to HCV treatment in SSPs. We also used expert opinion and personal knowledge to include additional literature if it met inclusion criteria and was not found with the established search terms used:

1. PubMed: (syringe services program OR syringe exchange program OR needle exchange program) AND (“hepatitis treatment”[tiab:∼3] AND “hepatitis c”)
2. Embase: (“syringe services program”/exp OR “syringe services program” OR “syringe exchange program”/exp OR “syringe exchange program” OR “needle exchange program”/exp OR “needle exchange program”) AND (“hepatitis c” NEAR/3 treatment)
3. Web of Science: (“syringe services program” OR “syringe services program” OR “syringe exchange program” OR “syringe exchange program” OR “needle exchange program” OR “needle exchange program”) AND (“hepatitis c” NEAR/3 treatment)

### Eligibility Criteria

Eligibility criteria included papers that were (a) written in English, (b) published after 2014, when all-oral regimens were approved in the United States, (c) involved SSPs that offer harm reduction supplies, and (d) described the complete continuum of HCV treatment. Protocol papers were included if there was an accompanying paper that included data. When multiple papers were identified that described the same program, the paper that most comprehensively described the program and its outcomes was included. Papers were excluded if they met the following criteria: (a) not US based, (b) limited to describing HCV testing and linkage to care but did not include integrated complete HCV care and harm reduction services. While we excluded programs that did not offer the complete continuum of HCV care through the SSP, an exception was made for programs that partnered with commercial laboratories for clinical evaluation.

### Selection Process

The articles produced by the search terms listed above were exported from the respective database and uploaded to a Microsoft Excel spreadsheet. Using the eligibility criteria above as a guide, 2 independent reviewers reviewed each article. If agreement to include the paper was reached, the article was uploaded to a separate spreadsheet. If there was disagreement, additional reviewers evaluated the article until a consensus was reached.

### Data Extraction and Analysis

The relevant data were extracted from the included articles using a matrix with the following categories: (a) study design, (b) setting and study population, (c) provider characteristics, (d) pretreatment evaluation, (e) medications, (f) funding, (g) supportive services, (h) treatment outcomes, and (i) current status of the program, if known. Key themes and trends were then summarized and categorized into major approaches to elements of the HCV care continuum.

## RESULTS

### Study Designs and SSP Settings

**Figure 1.**
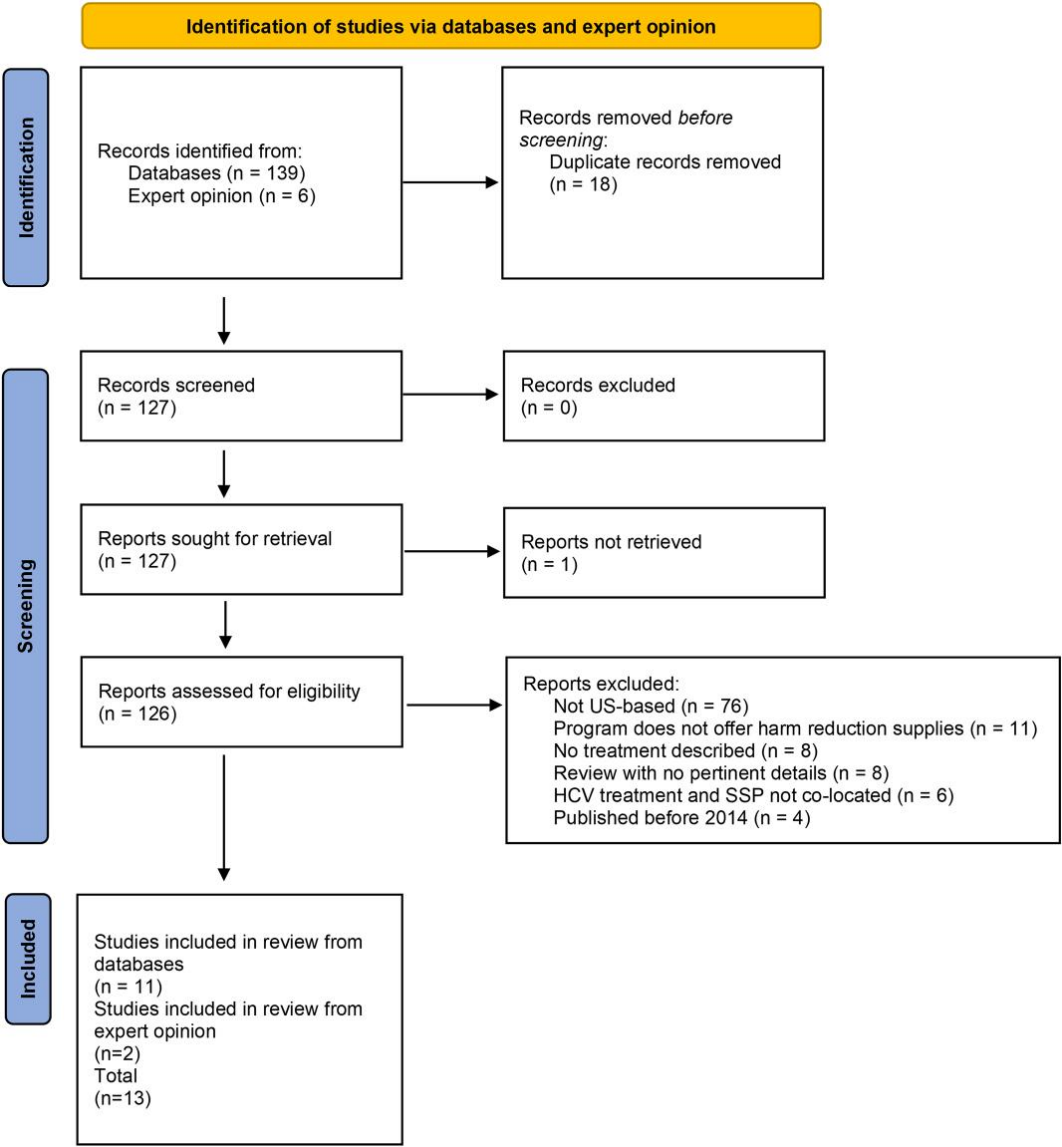
PRISMA flow diagram of the literature review. Abbreviations: HCV, hepatitis C virus; SSP, syringe service program.

The literature search yielded 128 articles, 13 of which met the eligibility criteria (Figure 1). Six papers described randomized controlled trials, 3 of which only described study protocols, while 3 described results of completed studies [20, 22–26]. One paper reported on a qualitative analysis of participant experience with HCV treatment through a program offering both primary care and needle and syringe services [27]. The remaining 6 papers included 1 retrospective review [28], 2 observational pilot studies [29, 30], and 3 cohort analyses [31–33] (Table 1). Eleven papers described urban programs, and 2 described rural programs. Most programs (8; 61%) utilized only brick-and-mortar SSP locations, 2 utilized only mobile SSP programs, and 3 programs offered both brick-and-mortar and mobile SSP services.

**Table 1.** Summary of Eligible Studies: Key Characteristics and Results

	Reference	Study Design	Location	Type of SSP	Other Services Offered	Provider Location	Study Results
1	Winetsky et al., 2020	Retrospective chart review	New York City, New York	Brick and mortar	Medication-assisted treatment, wound care, mental health services	On-site	SVR achieved in 89.6% of participants initiated on treatment (SVR4 vs 12 not specified)
2	Sivakumar et al., 2021	Cohort analysis	New Haven, Connecticut	Mobile & brick and mortar	STI treatment, MOUD, PrEP, HIV testing, and treatment	Telehealth	SVR12 achieved in 93.5% of participants initiated on treatment
3	Rosecrans et al., 2022	Cohort analysis	Baltimore, Maryland	Brick and mortar	MOUD, STI treatment, HIV testing and treatment, naloxone distribution	On-site via mobile health clinic outside SSPs	SVR12 documented in 42% of those initiated on treatment, 58% were lost to follow-up before SVR12 labs could be drawn
4	Eckhardt et al., 2022	Randomized controlled trial	New York City, New York	Brick and mortar	MOUD, HIV screening, overdose prevention training	On-site	SVR12 achieved in 67% of enrolled participants in the co-located arm compared with 23% of enrolled participants in the standard-of-care arm, SVR achieved in 85.9% of participants who initiated treatment in the co-located arm compared with 86.3% of participants who initiated treatment in the standard-of-care arm
5	Eckhardt et al., 2022	Randomized controlled trial	New York City, New York	Brick and mortar	MOUD, HIV screening, overdose prevention training	On-site	SVR12 achieved in 64.3% of enrolled participants in the rapid start, colocated treatment arm compared with 9.1% of enrolled participants in the referral treatment arm, SVR12 achieved in 92.3% of participants who initiated treatment in the rapid start, colocated treatment arm compared with 33.3% of participants who initiated treatment in the referral treatment arm
6	Eckhardt et al., 2018	Cohort analysis	New York City, New York	Brick and mortar	Comprehensive addiction treatment	On-site	SVR12 achieved in 91% of those who initiated treatment
7	Williams et al., 2019	Qualitative analysis	Portland, Oregon	Brick and mortar and mobile	Primary care	On-site	“Life projects” including social redemption, strengthening of relationships, pursuit of abstinence from substance use, and harm reduction motivated participants to complete HCV treatment
8	Martel-Laferriere et al., 2022	Randomized controlled trial	Miami, Florida	Brick and mortar and mobile	MOUD, wound care, safe injection packs, and naloxone packs	On-site	Pending
9	Klaman et al., 2024	Interventional comparative effectiveness trial	San Diego, California	Mobile	HIV testing	On-site medical mobile unit adjacent to mobile SSP	Pending
10	Seaman et al., 2021	Open-label pilot study enrolling patients with HCV at SSP and OTP	Portland, Oregon	Brick and mortar	Basic health care, reproductive health, HIV/STI testing, behavioral health, dental screening, MOUD, pharmacy	On-site	SVR12 achieved in 60% of patients treated at SSP compared with 96% in OTP in intent-to-treat analysis of those who initiated therapy, and 88% treated at SSP compared with 100% in OTP in the modified per-protocol SVR12 (those who initiated therapy and completed SVR confirmation labs)
11	Bianchet et al., 2024	Protocol commentary of randomized controlled trial	Rural Vermont and New Hampshire	Mobile	No additional services offered besides harm reduction and HCV testing and treatment	Telehealth	Pending
12	Seaman et al., 2024	Randomized controlled trial	Rural Oregon	Brick and mortar	None mentioned	Telehealth	SVR12 achieved in 63% of those who enrolled in the telehealth arm compared with 16% enrolled in the referral arm SVR12 74% among those in the telehealth arm who initiated treatment
13	Tsui et al., 2024	Prospective observational pilot study	Seattle, Washington	Brick and mortar	PrEP, naloxone, STI treatment	On-site	SVR12 achieved in 91% of those completing SVR testing

Abbreviations: HCV, hepatitis C virus; MOUD, medications for opioid use disorder; OTP, opioid treatment program; PrEP, pre-exposure prophylaxis; SSP, syringe services program; STI, sexually transmitted infection; SVR, sustained virologic response; SVR4, sustained virologic response 4 weeks after the end of treatment; SVR12, sustained virologic response 12 weeks after the end of treatment.

### Prescribing Clinician

In the majority of studies (n = 10), the clinician and patient interacted face-to-face through encounters on mobile medical units parked adjacent to the SSP (2 programs) or on-site within the mobile SSP unit or SSP facility (8 programs). However, 3 programs conducted clinician visits solely by telehealth. The prescriber was a physician or nurse practitioner in all but 1 of the studies, which described an on-site prescribing clinical pharmacist supervised by an off-site physician via collaborative practice agreement [30].

### Pretreatment Evaluation

Laboratory specimens for the majority of the studies were collected via on-site phlebotomy at the SSP and transported to a local laboratory for testing. Two studies required patients to go to a commercial laboratory for pretreatment evaluation labs [25, 31]. Most programs required a visit with a clinician for pretreatment assessment including ordering laboratory studies, although 1 paper described standing orders for all pretreatment laboratories so a provider visit to order laboratory tests was not necessary [25].

All studies required HCV RNA results before starting direct-acting antiviral (DAA) medications, although 1 study dispensed 1-week starter packs while awaiting results and instructed patients not to start the medications until the results were available [26]. Four studies tested for HCV genotypes, 2 of which limited participation according to genotype. Six studies described HIV testing as a required pretreatment element, and 6 studies described hepatitis B (HBV) surface antigen testing; the others did not mention HIV or HBV testing.

Pretreatment noninvasive fibrosis assessments were most frequently accomplished through assessment of routine liver tests and platelet counts that were used to calculate FIB-4 scores [34]. Two studies mentioned use of serum biomarker testing (FibroSURE), and 1 study required elastography (FibroScan) that was available on-site at the mobile treatment unit adjacent to a mobile SSP. Seven programs excluded participants with evidence of decompensated cirrhosis, 3 studies excluded participants with any cirrhosis, 1 study excluded participants with fibrosis stage 3 or greater, and 2 papers did not include information about exclusion based on fibrosis status. Four programs excluded individuals with HIV, and 1 program excluded individuals with HBV. No studies excluded people for drug use.

### Provider Visits, Prescribing, and Pharmacy Protocols

**Figure 2.**
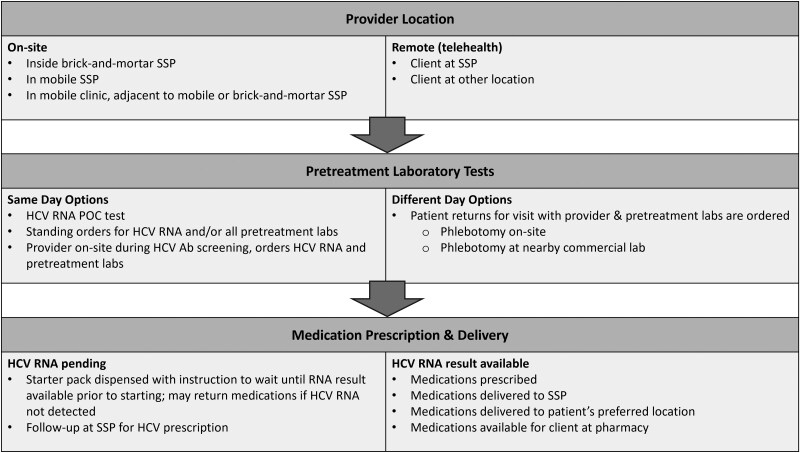
Approaches to elements of HCV treatment in SSPs. Common treatment pathways among programs. Appointments varied from walk-in to scheduled. Abbreviations: Ab, antibody; HCV, hepatitis C virus; POC, point of care; SSP, syringe service program.

A variety of approaches to treatment were used (Figure 2). Programs that did not have providers on-site at the time of antibody testing assessed HCV RNA in various ways. Some programs used standing orders to collect pretreatment laboratory tests for individuals with positive HCV antibody results, while others connected individuals to a provider before additional laboratory testing. If the HCV RNA result was not available yet, typically 1 provider visit was needed to order follow-up laboratory testing, followed by a second appointment, either drop-in or scheduled, to discuss treatment if HCV RNA was positive. One program gave all patients with positive HCV antibodies a 1-week supply of DAAs and instructed patients to wait for the HCV RNA results before starting medication; if HCV RNA was not detected, patients were instructed to return the 1-week starter pack [26]. Appointments were most commonly located at the SSP or at a mobile clinic parked in proximity to the SSP. Drop-in appointments included flexible hours and a consistent schedule for when the clinical prescriber would be available as well as care managers who helped schedule appointments if needed. Treatment regimens were most often glecaprevir/pibrentasvir or sofosbuvir/velpatasvir, although other regimens—elbasvir/grazoprevir, sofosbuvir/ledipasvir, and sofosbuvir/velpatasvir/voxilaprevir—were also described. For the majority of the programs, monthly appointments were scheduled with walk-in options to evaluate medication adherence and any adverse effects.

Medications were most often delivered to the patient's home or SSP (n = 11) or picked up at a local pharmacy (n = 2). Most programs offered options for patients to pick up medications at the SSP, where there were options for medication storage until patients could pick medications up at their convenience. One program delivered medications by scooters or bicycles [22]. For most programs, patients could decide what dispensing schedule worked best for them, with the options ranging from daily to weekly or monthly. Follow-up appointments during treatment to check in were frequently offered, and all programs assessed patients for cure using SVR12.

### Funding

All of the programs were dependent on grant funding for various elements. Funding sources included federal research programs; federal, state, and local public health funds; pharmaceutical companies; various foundations; and grant programs from the authors' respective institutions. Medical insurance was utilized for medication coverage in 7 of 13 programs, 4 programs offered free medications, and 2 did not specify how HCV medications were obtained. Additionally, some programs mentioned helping qualified uninsured patients enroll in pharmaceutical assistance plans.

### Supportive Services

Each program involved supportive services provided by peer navigators, case managers, or outreach coordinators. These services included facilitating drop-in or scheduled appointments, counseling, treatment education, insurance enrollment, managing medication delivery, and supporting adherence. Additionally, support staff offered education about HCV and reinfection risk, connected patients with community resources, disclosed testing results, and created personalized adherence plans. These individuals were employees from an affiliated academic health center (n = 4), were employed by the SSP, or were identified through various community organizations or word-of-mouth as potential peer navigators. The selection process for these support positions prioritized applicants who were nonjudgmental, empathetic, and had a common goal of dismantling stigma for those who use drugs. For at least 2 of the programs, these individuals worked full time for the program and did not have competing employment priorities. Additional supportive services offered by the programs included contingency management [35], which provided participants with $10–$70 gift cards for scheduling and attending follow-up appointments at the SSP to help confirm SVR12. No programs offered financial incentives for initiating treatment.

### Treatment Outcomes

Nine studies, with sample sizes ranging from 25 to 102 patients, reported outcomes with varying degrees of detail. Four studies reported SVR12 rates for participants enrolled in the study (63% to 67%) and for those who initiated treatment (42% to 100%). Seven studies reported DAA initiation (33% to 93%), and 6 studies reported treatment completion of those initiating treatment (54% to 94%). Of the 3 RCTs, SVR12 rates were reported to be superior in the treatment arms describing integrated care (63% to 67%) of participants enrolled in the study compared with arms where patients were referred externally for care (9.1% to 23%). The 6 observational studies reported SVR12s ranging from 42% to 93.5%.

### Current Program Status

Three programs had not yet reported study outcomes at the time of publication [23, 24, 26]. Of the remaining 10, corresponding authors for 8 studies confirmed ongoing HCV treatment with support provided through various funding sources, including city and state public health departments, grants from the Centers for Disease Control Viral Hepatitis Surveillance and Prevention, the Substance Abuse and Mental Health Services Administration, and private foundations. One program fully integrated HCV treatment with other clinical care offered that utilizes insurance billing, and 1 program did not have continued funding and was no longer providing HCV treatment.

## DISCUSSION

In this scoping review, we summarize the published findings of 13 programs that offered HCV treatment for PWID outside of the traditional clinic-based care model. The programs, which included in-person appointments or mobile units adjacent to SSPs, as well as telehealth-based care, allowed for flexibility in care delivery for both the participant and the provider. Pretreatment evaluations primarily involved on-site phlebotomy at SSPs followed by delivery of DAAs directly to SSPs or other preferred locations, with on-site medication storage and adjustable dispensing schedules. Supportive services, including peer navigators, case managers, enrollment assistance, and contingency management, facilitated engagement in care and medication adherence.

All programs described a process for obtaining blood for pretreatment laboratory assessments and required a detectable HCV RNA result before starting treatment. Most programs required noninvasive laboratory-based fibrosis assessments, as well as HIV and HBV testing. Most sites described on-site phlebotomy while 1 program described a team member accompanying clients to a commercial laboratory with pre-approval from the program's medical director for phlebotomy from lower extremity veins if needed [36]. The importance of a skilled, highly experienced phlebotomist on site at the SSPs was emphasized in multiple papers and is another potential benefit of the SSP-based care model [37].

While SVR12 outcomes were not available for all programs at the time of publication, the programs that did include final results reported cure rates ranging from 54% to 94% of those who enrolled, initiated therapy, and/or completed SVR12 confirmatory labs, depending on the study. SVR12 results and treatment initiation rates in the studies reviewed were similar to outcomes reported from international SSP-based HCV treatment programs, which consistently report significant improvements compared with standard-of-care approaches [14–16].

These results demonstrate how SSPs have trusted advocates that serve a highly marginalized community of PWID and are essential public health partners in achieving HCV elimination goals [38]. They typically offer various supportive services and build longitudinal relationships with participants, facilitating opportunities for clinical care to individuals who may not otherwise engage with health care providers [39]. Integration of HCV services where PWID actively receive harm reduction services has been shown to address barriers to treatment at all levels of the care continuum [40, 41] and to be responsive to PWID preferences [15]. Telehealth services, more widely used for HCV treatment since the COVID-19 pandemic [31, 40], allow for access to medical providers from the comfort of a familiar SSP setting. Importantly, facilitated telehealth has also been shown to increase HCV treatment rates for people with opioid use disorder in methadone treatment programs [42]. However, HCV treatment program sustainability has been limited by various barriers including timely treatment initiation, lab monitoring logistics, and variable funding sources [43].

Although most programs aimed to initiate treatment at the second visit and/or within 2 weeks of screening, Eckhardt et al. demonstrated that rapid initiation with a 7-day starter pack provided on the day of screening was an effective strategy to achieve SVR12. Implementation of same-day medication starts is a promising approach to promote engagement and increase efficiency, although it would not be feasible without donated medications or another nontraditional procurement method, grant funding, and/or local public health support.

With the 2024 FDA approval of point-of-care HCV RNA testing that provides results in about an hour, programs may be able to offer a same-day “test and treat” strategy; however, the cost of the technology and required infrastructure for device utilization are likely to present substantial practical barriers to large-scale implementation [44]. With the introduction of generic DAAs in the coming years, the price of HCV treatment may decrease substantially, increasing the feasibility of same-day treatment options [45]. Notably, other elements of the pretreatment evaluation, such as HBV and HIV screening and FIB-4 fibrosis score, still require phlebotomy for laboratory-based testing, further impacting the plausibility of same-day treatment initiation [46].

Whether treatment should be delayed while waiting for results is a clinical decision that programs must consider. National guidelines for HIV pre-exposure prophylaxis suggest that “same-day starts” while awaiting laboratory results are safe and effective, stipulating that obtaining patient contact information is critical in case resultant labs necessitate a change to management [47]. Of note, at the time of this publication, the American Association for the Study of Liver Diseases and the Infectious Diseases Society of America joint HCV guidelines committee approved a similar test and treat algorithm, with special consideration to initiation of HBV treatment and referral to specialty care if HBV surface antigen testing is positive after HCV treatment initiation [48]. While this strategy would greatly shorten HCV treatment initiation, limited contact information or follow-up plans for reviewing results could pose serious logistical and medical challenges, particularly given the high rates of homelessness among PWID [49]. Although ∼1.4% of all individuals with HCV in the United States are coinfected with HBV [50], this may be a more common problem among PWID who are at enhanced risk of coinfection [51]. Weighing the potential individual clinical and larger public health benefits of HCV treatment with the risks of HBV viral reactivation during HCV treatment must be included in treatment counseling and patient-centered decision-making [52]. Beyond considering HCV treatment initiation before completed laboratory results, the costs and logistics of managing these additional tests pose challenges to program sustainability.

Establishing consistent funding for integrated HCV programs is challenging in the US health care system. Of the 9 states represented in this review, 8 had expanded Medicaid access at the time of publication with varying eligibility criteria for DAA coverage [53]. As Medicaid programs become less restrictive [54], greater access to HCV treatment is becoming a reality for many PWID, but treatment in nonexpansion states remains a considerable obstacle. Programs that did not use the participants' insurance were largely funded by grants from the study sponsors, often with pharmaceutical companies providing medication. Outside of clinical trials, pharmaceutical assistance plans can be an option for some uninsured individuals, although access to these plans is time-intensive and requires key financial documents that are impractical at best and impossible for many, especially PWID with unstable housing. The plausibility of continuing these programs beyond temporary or limited grant funding is contingent upon identifying more sustainable sources of funding than currently available.

Even with Medicaid expansion, reimbursement rates for provider visits and laboratory tests may not meet the costs incurred [55]. An economic analysis by Eckhardt et al. found that start-up, overhead, and clinical coordination costs far exceeded potential insurance reimbursement. To initiate and sustain successful programs, improved reimbursement rates or other public sources will be critical due to the inherent instability of continued grant support. Funding from public health departments or private foundations has allowed for sustainable SSP-based HCV care in several of the programs we reviewed.

In terms of setting, only 2 out of the 13 articles reviewed were located in rural environments [24, 25]. Rural individuals are particularly vulnerable to rapidly increasing opioid use, overdose, and infectious disease transmission [56, 57]. Community assessments have shown that PWID in rural locations are discouraged from participating in harm reduction services due to concerns about stigma from their communities, mistrust of institutions, limited availability of programs, poor transportation infrastructure, and criminalization of drug use, among other barriers [58, 59]. In the context of these obstacles, interventions for rural settings must be designed with consideration for this population's unique needs and socioeconomic realities. Bianchet and Seaman addressed rural specific barriers by employing mobile units and peer navigators; however, these intervention strategies have not been as robustly studied in rural settings compared with urban environments. Thus, increased exploration of nonurban HCV treatment interventions is an essential direction for future investigation and investment into HCV elimination.

Further research on these models may include qualitative and quantitative assessment of treatment priorities of PWID in SSP-based HCV treatment in order to ensure that programming is aligned with patients' needs and enhanced by stronger assessment models. Additionally, advocating for expansive public health funding of harm reduction measures in general would alleviate funding barriers toward HCV treatment in these settings. The successful cure rates seen in these programs may also demonstrate promising treatment and prevention strategies for other bloodborne infectious diseases such as HIV, HCV, or other STIs.

## CONCLUSIONS

We describe 13 unique programs situated in SSPs in the United States that offered the full continuum of HCV treatment from diagnosis to cure. These programs differed in their approaches to provider location, pretreatment evaluations, medication delivery and storage, and wrap-around services, but all described close collaboration with partnering SSPs as crucial to their success. Treatment initiation, completion, and SVR12 rates of the SSP-based HCV treatment programs were variable but all demonstrated marked improvements in access to care compared with current baseline levels of HCV treatment among PWID. Despite the overall success of these models, substantial challenges to sustainability and scalability remain. Funding for program implementation and sustainability is the most pressing challenge, along with treatment coverage for uninsured individuals. Modest public health funding for these impactful programs coupled with treatment coverage through public insurance or cost assistance programs could enable substantial scale-up of these approaches, harnessing the benefits of SSPs not only for the goal of eliminating HCV, but also as an entry to a broader range of key health care services.
